# Editing of *BoMLO12* confers broad-spectrum disease resistance and enhanced yield in *Brassica oleracea*

**DOI:** 10.1093/hr/uhag009

**Published:** 2026-01-09

**Authors:** Yulun Zhang, Guanzhong Huo, Limei Yang, Yangyong Zhang, Mu Zhuang, Yong Wang, Jialei Ji, Xuehui Yao, Honghao Lv

**Affiliations:** State Key Laboratory of Vegetable Biobreeding, Institute of Vegetables and Flowers, Chinese Academy of Agricultural Sciences, Beijing 100081, China; State Key Laboratory of Vegetable Biobreeding, Institute of Vegetables and Flowers, Chinese Academy of Agricultural Sciences, Beijing 100081, China; State Key Laboratory of Vegetable Biobreeding, Institute of Vegetables and Flowers, Chinese Academy of Agricultural Sciences, Beijing 100081, China; State Key Laboratory of Vegetable Biobreeding, Institute of Vegetables and Flowers, Chinese Academy of Agricultural Sciences, Beijing 100081, China; State Key Laboratory of Vegetable Biobreeding, Institute of Vegetables and Flowers, Chinese Academy of Agricultural Sciences, Beijing 100081, China; State Key Laboratory of Vegetable Biobreeding, Institute of Vegetables and Flowers, Chinese Academy of Agricultural Sciences, Beijing 100081, China; State Key Laboratory of Vegetable Biobreeding, Institute of Vegetables and Flowers, Chinese Academy of Agricultural Sciences, Beijing 100081, China; State Key Laboratory of Vegetable Biobreeding, Institute of Vegetables and Flowers, Chinese Academy of Agricultural Sciences, Beijing 100081, China

Dear Editor,

*Brassica oleracea*, a biennial herbaceous species within the *Brassicaceae* family, includes a variety of globally cultivated vegetable crops of significant agronomic and economic importance, such as cabbage, broccoli, and kale [1]. However, their production is severely constrained by soil-borne and foliar diseases, such as *Fusarium* wilt caused by *Fusarium oxysporum* f. sp. *conglutinans*, black rot caused by *Xanthomonas campestris* pv. *campestris*, and black spot caused by *Alternaria brassicicola*.

Disease-resistant cultivars are typically developed by introducing dominant resistance (*R*) genes and knocking out susceptibility (*S*) genes. Notwithstanding these approaches, *R* genes typically confer resistance solely to specific cognate pathogens, thereby limiting broad-spectrum resistance. At the same time, editing *S* genes sometimes confers plants with nonspecific, durable, and broad-spectrum disease resistance. Thus, harnessing loss-of-function mutations in *S* genes has emerged as a promising strategy for breeding disease-resistant crops. The advent of Clustered Regularly Interspaced Short Palindromic Repeats/CRISPR-associated protein (CRISPR/Cas) systems has revolutionized plant genome engineering, enabling precise genetic modifications and significantly facilitating the development of crop varieties with enhanced disease resistance [2]. According to previous studies, editing the *BoBPM6* and *BoDMR6* genes resulted in the creation of new germplasms with broad-spectrum disease resistance in cabbage [3]. In addition, *MLO* is a well-characterized *S* gene, and the wheat *MLO* mutant *Tamlo*-*R32* has been demonstrated to exhibit broad-spectrum resistance against powdery mildew while maintaining optimal yield potential [4].

In this study, we conducted functional characterizations of the *MLO* genes in cabbage (*B. oleracea* var. *capitata*)*.* To identify potential target for enhancing disease resistance in *B*. *oleracea*, phylogenetic analysis was conducted on 28 identified *MLO* homologs. Among these, 22 genes containing complete functional motifs were selected for phylogenetic tree construction with *Arabidopsis thaliana MLO* genes, indicating *BoMLO12* (*Bo4g188000.1*) as a promising candidate. Notably, *BoMLO12* showed high sequence similarity to *Tamlo-R32* (Fig. 1A). Subsequently, a specific target site within the fourth exon of *BoMLO12* was edited using CRISPR/Cas9 technology (Fig. 1B and C). Through *Agrobacterium*-mediated transformation, a transformation efficiency of 4.6% and an editing efficiency of 42.0% (3/7) were attained. Additionally, Sanger sequencing of T_1_ generation *bomlo12* mutants (*bomlo12-1*, *bomlo12-2*, *bomlo12-3*) revealed six distinct mutation types, including insertions, deletions, and substitutions (Fig. 1C). Based on genetic characterization, inoculation assays were performed using three distinct pathogens: root dipping method for *Fusarium* wilt, needle puncturing method for black rot, and mycelial plug inoculation method of black spot. Three independent T_2_  *bomlo12* mutant lines were subjected to standardized pathogenicity assays. Comparative disease assessment revealed that *bomlo12* mutants exhibited statistically significant reductions in disease index (DI) values compared to wild-type (WT) controls, from 31.4 to 21.8 (significant) for black rot, from 65.4 to 15.3 (significant) for *Fusarium* wilt, and from 77.8 to 43.3 (significant) for black spot (Fig. 1D–F). Subsequently, we performed quantitative real-time PCR of the *BoMLO12* and defense-related genes in both WT and mutant plants before and after inoculation. The results demonstrated that the expression of *BoMLO12* gene showed a significant downregulation trend following inoculation. Similarly, the expression of the defense-related gene *BoPR10* was significantly downregulated, while both *BoPAL* and *BoNPR1* exhibited significant upregulation, which is consistent with previous research findings [5] (Fig. 1M and P).

**Figure 1:**
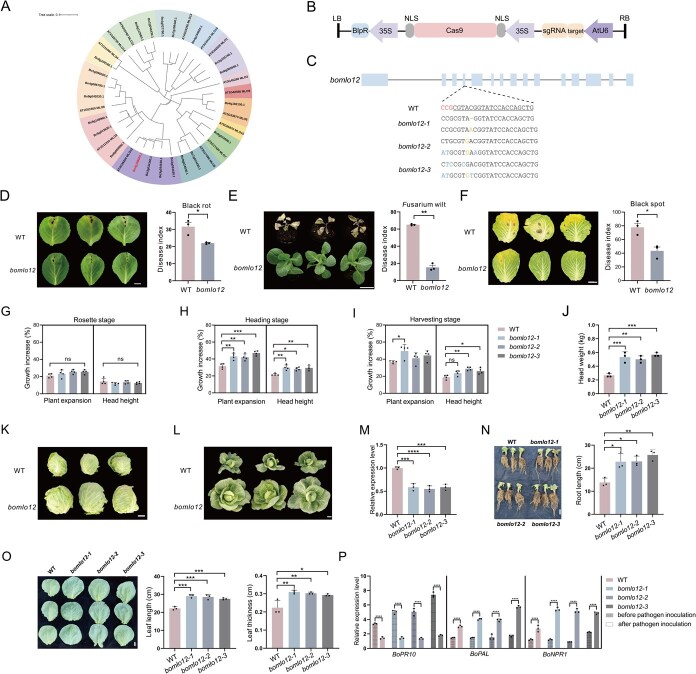
CRISPR/Cas9-mediated *BoMLO12* editing enhances disease resistance and agronomic performance in *B. oleracea.* (A) Phylogenetic relationship of *MLO* homologs in *B. oleracea* and *A. thaliana*. (B) Schematic of CRISPR-Cas9 vector design targeting *BoMLO12*. (C) Diagram of *BoMLO12* gene structures. Rectangular blocks represent the gene coding region; lines indicate intronic regions; dashed line illustrate the single guide RNA (sgRNA) target region (Target 1). The sequences of the WT and mutants are outlined below. The target sequence is underlined. The protospacer adjacent motif (PAM) sequence is highlighted in red, insertions in yellow, substitution in blue, and deletions marked with horizontal lines. (D–F) Phenotype and disease index of black rot, *Fusarium* wilt, and black spot in WT and knockout (*bomlo12*) plants. (G–I) Agronomic analysis of plant expansion and head height in WT and *bomlo12* plants across three different stages. (J, K) The head weight of WT and *bomlo12* plants. (L) Spread phenotype of WT and *bomlo12* plants in harvestng stage. (M) The expression levels of *bomlo12* were assessed in WT and knockout plants. (N, O) Investigation of agronomic traits in mutants, including taproot length, maximum outer leaf length, and leaf thickness. (P) The relative expression levels of defense-related genes were analyzed in the mutants before and after pathogen inoculation. For the disease inoculation tests, three knockout lines are selected for *bomlo12.* The whole inoculation experiment was repeated three times with similar results. ^*^P < 0.05; ^**^P < 0.01; ^***^P < 0.001; ^****^P < 0.0001 indicate significant differences determined by two-tailed Student’s *t*-test. Scale bars: 5 cm.

To evaluate the agronomic effects of *BoMLO12* knockout, three T_2_ generation *bomlo12* mutant lines and WT plants were grown under controlled conditions. We conducted biweekly field measurements of yield-related traits in both WT and *bomlo12* plants across three developmental stages: rosette stage, heading stage, and harvesting stage. During the rosette stage, *bomlo12* mutants showed a 10.7%–21.7% increase in plant expansion, with no significant change in head height observed. During the heading stage, compared to WT plants, all *bomlo12* mutants (*bomlo12-1*, *bomlo12-2*, and *bomlo12-3*) exhibited significant increases, with plant expansion rising by 32.8%–48.6% and head height by 30.6%–38.2%. During the harvesting stage, *bomlo12-1* displayed a significant 34.7% increase in plant expansion, while *bomlo12-2* and *bomlo12-3* showed significant head height increases of 38.4%–52.3% (Fig. 1G–I, L). Following this, outer leaves were removed to assess the fresh weight of individual heads. Notably, the individual head weight of *bomlo12* mutants was ~2-fold greater than that of WT plants (Fig. 1J and K). And the results of root growth (maximum root length) and plant growth parameters (leaf thickness, leaf length, and plant expansion index), also demonstrated statistically significant upward trends (Fig. 1N and O). These findings suggest that *BoMLO12* knockout enhances resistance to black rot, *F*usarium wilt, and black spot while improving agronomic performance in *B*. *oleracea* through increased plant size.

In summary, CRISPR/Cas9-mediated editing of the *BoMLO12* gene successfully produced novel *B*. *oleracea* germplasms, demonstrating enhanced broad-spectrum resistance and significantly improved yield performance. These findings establish a foundation for breeding *B*. *oleracea* varieties with increased disease resistance and productivity.

## Data Availability

The authors confirm that the data supporting the findings of this study are available within the article.
